# Abortion in an Hour

**Published:** 1892-06

**Authors:** Charles H. Harris

**Affiliations:** Cedartown, Ga.


### ABORTION IN AN HOUR.

BY CHARLES H. HARRIS, M. D., CEDARTOWN, GA.

The difficulties encountered in the treatment of abortion are so well known that is unnecessary to name them. The experienced physician has met with these cases in every stage of developement, from the neurotic prodromata to the emptying of the uterus and its subsequent involution. Mild forms of the accident rarely come under our observation as they transpire without exciting any alarm; women do not take their beds for them; indeed, it sometimes happens that they are themselves unconscious of their condition until they notice that "something has passed" from them.

As a rule, it is only the bad cases that call on us, such cases as are attended with great suffering or danger. There is a reason for this tardiness on the part of the women in seeking our aid. Reduced to its last analysis it means our objectionable management of them. We do not please them in this line of practice. The older doctors are painfully aware of this fact. They call upon us for relief and we do what we can—perhaps tent the cervix and tampon the vagina and tell them to be patient, that in due time everything will happen all right, or if the case will admit of it, we rapidly dilate, curette and extract as best we can with the usual instruments. It is this dallying conservatism and rough handling with uncertain results, too well known to the women, that keep them aloof from us until they are driven in from the desperation of fear. Is it any wonder when we review the results of our work! The death roll and wrecks of womanhood along the line of our march, justify their evasion of us, and we admire their wisdom. Along with their other attractions, the women have sense and they use it. Had we impressed them with our skill, things would have been different. Did they know that we could relieve them in a few moments or at most in an hour, we would gain their confidence, and the field of accidental abortion, as yet barely touched, would be fairly ours. That any case of abortion in which an operation is admissible, may be brought to a finish in an hour and on the first visit, I deem not only possible, but eminently practicable. If we find the cervix dilated or dilatable, a few minutes will suffice. Should we find it rigid, narrow and unyielding (a rare exception) it requires more time, patience and perseverance to overcome this difficulty—not however more than an hour. This contingency is met as we will directly understand by means similar to the obstetric forceps and the dilation goes on *pare passu* with the processes of detachment and expulsion. The instruments used are not forceps and dilators; they are combination curettes and snares made expressly for the operation and are adjustable in utero. They are of steel round-wire and flat stout watch-spring. With little difficulty they may be made to pass the cervix of any pregnant uterus and at the will of the operator project inside of the organ a curette or snare suited to the work. They are so slender as to practically occupy no space at all since they become embedded in the mass of the ovum. Having observed the rules of antisepsis, the operator passes the smallest loop of snare number 1 fairly within the uterus. This done (with average skill) he should be master of the situation. If nervous, the patient should have been chloroformed and in Sims' position. His task now is almost purely mechanical, viz., to detach, chop up into small fragments and remove the ovum. This is done by curetting and snaring. Curetting requires small loops of wire and should be thorough and rapid. Snare No. 1 having been passed into the cavity of the uterus, its loop is now enlarged an inch (twice the size of the thumb nail) and the instrument is urged on to its work of destruction. The attachments of the placenta are rapidly severed and the staff of the snare made to sweep around the globe of the ovum. To make assurance doubly sure, enlarge the loop another inch and scrape the uterus as you would a mortar with a thin spatula. Should the membranes survive this ordeal, they should now be ruptured. Draw the wires back through the staff so as to make a loop at its terminal end in the uterus. Now aim the small loop towards the center of the ovum and plunge it into the mass. Failing in this, snare No. 2 with small loop should be passed into the uterus and the membranes are easily ruptured. A better instrument cannot be devised for this purpose.

An eminent gynæcologist says: "It is between a dull and sharp curette and will take the place of both in many operations." Having ruptured the membranes, the work of snaring begins. For snaring purposes, a large loop of round wire is best. A large loop of snare No. 1 is sprung in the uterus and is kept moving until it hangs upon the ovum. Now pull with forceps, movement and steadily increasing force. There will be several results from this traction; it is the best, surest and safest and quickest way of dilating the cervix. It is a perfect uterine tampon. It will either extract entire, or else divide the ovum reducing its bulk and avoiding the necessity of largely dilating the cervix. The finest results, however, from this traction is its remarkable effect on the uterus. Through reflex agencies beginning in the cervix, contractions follow and we invoke the best efforts at expulsion. It is this vigorous co-operation of the uterus which stimulates so closely the typical abortion of a healthy woman. Should the wire cut its way through the structures of the ovum, it should be re-introduced and the snaring continued until mass in the utero is reduced to small fragments and extracted.

The snares have a traction power of over an hundred pounds, which is amply sufficient for the work. To complete the toilet of the uterus, snare No. 2 with flat watch-spring is passed with small loop, enlarge the loop until it presses well on the uterine wall. Pressing upwards the snare is rotated backwards and forwards on its terminal axis, until it works smoothly. This scrapes off the residual placenta, which with the detritus of the ovum is removed by passing the loop into and out of the uterus until the fragments cease to appear in it. It only remains to swab out the organ with a styptic antiseptic and irrigate the vagina with sterilized water. In this operation the surgeon does all the work. Nothing is left to nature or accident. It applies to the first three months of pregnancy, before the structures of the fœtus become toughened and hardened by ossification. The author is well aware of the magnitude of the task he has undertaken. Reforms in medicine are slow, and when they assume the dignity of revolution, as in this operation, they are met with bristling opposition from every quarter. It has been our purpose to place the operation for abortion where it has never been, among the certainties of surgery. Along with this, we hope to capture a field of practice for the profession which as yet has barely been touched. A doctor will not permit a woman to remain in labor for days without relief, and it will be equally culpable when this operation is understood for him to allow a case of abortion to drag its weary course along through days and weeks, as heretofore. More than this, he will be held accountable, if he does not relieve his case on the first visit.

---

Have used Peacock's Bromides in my practice for some time, and I would not like to be without it, in fact I do not know of anything that would take its place in nervous conditions. J. T. KILBURN, M. D., Trufant, Mich.

